# OSU-6162, a Sigma1R Ligand in Low Doses, Can Further Increase the Effects of Cocaine Self-Administration on Accumbal D2R Heteroreceptor Complexes

**DOI:** 10.1007/s12640-019-00134-7

**Published:** 2019-11-28

**Authors:** Dasiel O. Borroto-Escuela, Wilber Romero-Fernandez, Karolina Wydra, Zilong Zhou, Agata Suder, Malgorzata Filip, Kjell Fuxe

**Affiliations:** 1 Department of Neuroscience, Karolinska Institutet, Stockholm, Sweden; 2 Department of Biomolecular Science, Section of Physiology, University of Urbino, Campus Scientifico Enrico Mattei, Via Ca’ Le Suore 2, 61029 Urbino, Italy; 3 Observatorio Cubano de Neurociencias, Grupo Bohío-Estudio, Zayas 50, 62100, Yaguajay, Cuba; 4 Science for Life Laboratory, Department of Cell and Molecular Biology, Uppsala, Sweden; 5 Maj Institute of Pharmacology Polish Academy of Sciences, Department of Drug Addiction Pharmacology, Kraków, Poland; 6 National Engineering Laboratory for Druggable Gene and Protein Screening, Northeast Normal University, Changchun, China

**Keywords:** A2AR-D2R heteroreceptor complexes, allosteric receptor-receptor interactions, cocaine use disorder, monoamine stabilizer

## Abstract

Cocaine was previously shown to act at the Sigma1R which is a target for counteracting cocaine actions. It therefore becomes of interest to test if the monoamine stabilizer (–) OSU-6162 (OSU-6162) with a nanomolar affinity for the Sigma1R can acutely modulate in low doses the effects of cocaine self-administration. In behavioral studies, OSU-6162 (5 mg/kg, s.c.) did not significantly change the number of active lever pressing and cocaine infusions. However, a trend to reduce cocaine readouts was found after 3 days of treatment. In contrast, in maintenance of cocaine self-administration, the proximity ligation assay performed on brains from rats pretreated with OSU-6162 showed highly significant increases in the density of the D2R-Sigma1R heteroreceptor complexes in the shell of the nucleus accumbens versus OSU-6162 induced increases in this region of yoked saline rats. In cocaine self-administration, highly significant increases were also induced by OSU-6162 in the A2AR-D2R heteroreceptor complexes in the nucleus accumbens shell versus vehicle-treated rats. Furthermore, ex vivo, the A2AR agonist CGS21680 (100 nM) produced a marked and significant increase of the D2R Ki high values in the OSU-6162-treated versus vehicle-treated rats under maintenance of cocaine self-administration. These results indicate a substantial increase in the inhibitory allosteric A2AR-D2R interactions following cocaine self-administration upon activation by the A2AR agonist ex vivo. The current results indicate that OSU-6162 via its high affinity for the Sigma1R may increase the number of accumbal shell D2R-Sigma1R and A2AR-D2R heteroreceptor complexes associated with further increases in the antagonistic A2AR-D2R interactions in cocaine self-administration.

## Introduction

The Sigma1R is known to be a cytoplasmic chaperone found in the endoplasmic reticulum in its interaction zone with the mitochondria (Kourrich et al. 2012). Cocaine can target the sigma1R upon which the Sigma1R is translocated into the surface membrane. Reaching the plasma membrane, it inter alia forms complexes with dopamine D1R and D2R and Kv1.2 ion channels and modulates their function (Borroto-Escuela, 2019; Kourrich et al. 2013; Navarro et al. 2010; Navarro et al. 2013; Pinton et al. 2015a; Pinton et al. 2015b). In addition, cocaine-induced changes in transcription of Sigma1R also takes place and preferentially in the ventral striatum with increased expression of Sigma1R (Romieu et al. 2002).

It should be noted that in acute experiments on D2R-Sigma1R heteroreceptor complexes in cells, cocaine increased the D2R recognition and signaling and markedly diminished the D2R internalization (Borroto-Escuela, 2019; Pinton et al. 2015a; Pinton et al. 2015b). In line with these results, it was observed in striatal synaptosomes that cocaine in acute experiments enhanced Gi/o-mediated signaling of D2R as found in studies on D2R and Sigma1R interactions in their modulation of dopamine and glutamate release (Beggiato et al. 2017).

In cocaine, self-administration marked changes in the multiple D2 heteroreceptor complexes developed in the ventral striatum, mainly in the nucleus accumbens shell (Borroto-Escuela et al. 2018b). The A2AR-D2R-Sigma1R complex appeared to become the dominant complex. In fact, highly significant antagonistic A2AR-D2R interactions were found in the ventral striatum associated with significant increases in A2AR-D2R and Sigma1R-D2R heteroreceptor complexes during cocaine self-administration (Borroto-Escuela et al. 2019; Borroto-Escuela et al. 2017; Pintsuk et al. 2016). The inhibition of cocaine self-administration by the A2AR agonist was blocked by a receptor interface interfering peptide (A2AR-TM5) disrupting the A2AR-D2R complex (Borroto-Escuela et al. 2018c). However, in cell lines expressing A2AR-D2R-Sigma1R heteroreceptor complexes, cocaine markedly enhanced the A2AR agonist-induced inhibition of Gi/o-mediated signaling of the D2R as studied in a CREB (cAMP response element-binding protein) assay (Borroto-Escuela et al. 2019).

In contrast, in the yoked-saline controls, no inhibitory A2AR-D2R interactions were found (Pintsuk et al. 2016). It may be explained by proposing that the Sigma1R present in the ventral striatum preferentially binds to the D2R homomers-monomers which may enhance the D2R signaling and counteract the consequences of limited antagonistic A2AR-D2R interactions. The molecular and cellular mechanism involved in cocaine self-administration was proposed to be a substantial increase in the formation of A2A-D2R-Sigma1R complexes mainly in the nucleus accumbens shell with restored or enhanced A2AR-D2R antagonistic receptor-receptor interactions. It was all made possible through the increased expression of Sigma1R mainly in the nucleus accumbens shell (Borroto-Escuela et al. 2019; Borroto-Escuela et al. 2018b).

The current study was performed to test the above hypothesis by studying the role of the Sigma1R in cocaine self-administration based on treatment with a low dose (5 mg/kg) of the monoamine stabilizer (–) OSU-6162 (OSU-6162 ) (Carlsson and Carlsson 2006; Carlsson and Carlsson, 2004; Feltmann et al. 2018; Steensland et al. 2012). In such a low dose, OSU-6162 will preferentially target the Sigma1R versus the D2R itself, since its affinity for the sigma1 receptor is in the nanomolar range (Sahlholm et al. 2013). Our studies also involved ex vivo analysis of the density of accumbal A2AR-D2R and D2R-Sigma1R heteroreceptor complexes using in situ proximity ligation assay (PLA) (Borroto-Escuela et al. 2019) and of allosteric A2AR-D2R receptor-receptor interactions induced by an A2AR agonist CGS21680 given ex vivo using biochemical binding techniques (Pintsuk et al. 2016).

## Materials and Methods

### Animals

Male Sprague-Dawley (derived from the licensed animal breeder Charles River, Sulzfeld, Germany), weighing between 250 and 270 g at the beginning of the experiment, is used. All animals used in the study were experimentally naive. The animals were housed individually in standard plastic rodent cages in a colony room maintained at 22 ± 1 °C and 55 ± 10% humidity under a 12-h light-dark cycle (lights on at 6:00 am). Rodent food (VRF1 pellets, UK) and water were available ad libitum except for the period of the initial training sessions when rats were maintained on limited water. All protocols were conducted during the light phase of the light-dark cycle between 9:00 and 13:00 hours. The experiments were carried out in accordance with the European Directive 2010/63/EU and were approved by the Ethical Committee at the Institute of Pharmacology, Polish Academy of Sciences, Krakow.

### Drugs

Cocaine hydrochloride ((1R, 2R, 3S, 5S)-3-(benzoyloxy-8-methyl-8-azabicyclo[3.2.1]octane-2-carboxylic acid methyl ester hydrochloride; **Toronto Research Chemicals (TRC)**, Canada) was dissolved in sterile 0.9% NaCl and administered *i.v.* in a volume of 0.1 ml per infusion. OSU-6162 hydrochloride ((3*S*)-3-[3-(methylsulfonyl)phenyl]-1-propylpiperidine hydrochloride; Tocris, UK, 0.1 mg/kg) was dissolved in 0.9% NaCl and administrated *s.c.* 60 min before 2-h self-administration session in a volume of 0.1 ml/kg.

### Surgery

Animals were anesthetized with ketamine HCl (75 mg/kg, *i.m.*; Biowet, Poland) and xylazine (5 mg/kg, *i.m.*; Biowet, Poland) cocktail and chronically implanted with a Silastic catheter in the external jugular vein, as described previously (Filip et al. 2006). During 3 days after surgery, meloxicam (*Metacam*, Boehringer Ingelheim; 5 mg/kg, *s.c.*) was used to reduce post-operative pain.

Rats were allowed 7–9 days to recover from surgery before the start of the experiments. Catheters were flushed daily with 0.2 ml of saline solution containing heparin (100 U/ml, Biochemie, Austria) and 0.1 ml of a cephazolin solution (100 mg/ml Biochemie GmbH, Austria) to prevent catheter non-patency. Catheter patency was tested periodically with the short-acting barbiturate anesthetic methohexital (10 mg/kg, *i.v*.), which induced the loss of consciousness within 5 s. No problems with catheter patency were reported in the tested rats.

### Initial Training (Lever Presses)

A day before lever presses training during three days of training, each rat had food limited to 25 g per day. Animals were trained for 3 days to press a lever (“active” lever) for 2 h daily in standard operant chambers (Med-Associates, St. Albans, GA, USA) under a fixed ratio (FR) from 1 to 5 schedule of reinforcement of sweetened milk.

### Cocaine Self-Administration

**Fig. 1:**
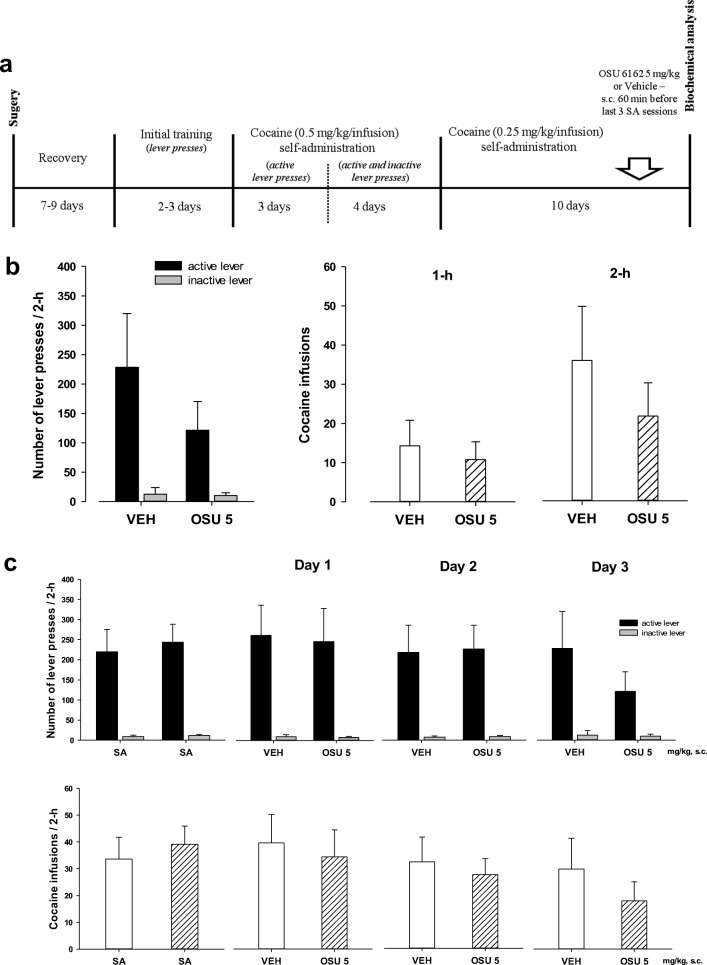
**(A)** Experimental design of the study. Schematic diagram illustrating the experimental procedure. SA – self-administration sessions. **(B)** Effects of OSU-6162 (OSU; 5 mg/kg, *s.c.*) or the corresponding vehicle (VEH; *s.c*.) treatments on the maintenance of cocaine (0.25 mg/kg/infusion) self-administration under the FR 5 schedule of reinforcement in the rats. The numbers of “active” and “inactive” lever presses as well as cocaine infusions after 1 and 2 h are expressed as the means (± SEM) of the data from 7 to 8 rats/group. **(C)** Effects of repeated administration of OSU-6162 (OSU; 5 mg/kg, s.c.) or the corresponding vehicle (VEH, 0.9% saline, s.c.) treatments on the maintenance of cocaine (0.25 mg/kg/infusion) self-administration under the FR 5 schedule of reinforcement in the rats. The numbers of active and inactive lever presses (upper panels) as well as cocaine infusions (lower panels) after 2 h are expressed as the means (± SEM) of the data from 7 to 8 rats/group. SA – mean of last three cocaine self-administration sessions

After the recovery period, all animals began lever pressing for cocaine reinforcement during 2-h daily sessions performed 6 days per week. The house light was illuminated throughout each session. Each press on the “active” lever (FR-5 schedule of reinforcement) resulted in a 5-s infusion of cocaine (0.5 mg/kg per 0.1 ml) and a 5-s presentation of a stimulus complex (activation of the white stimulus light directly above the “active” lever and the tone generator). Following each injection, there was a 20-s time-out period during which responding was recorded but had no programmed consequences. Presses on the “inactive” lever were recorded, but not reinforced. After the 7 days of acquisition, rats were used to complete a cocaine (0.25–0.5 mg/kg/infusion) dose-response curve. Cocaine self-administration was conducted daily for 17 sessions. Following stabilization of responding rates with cocaine (0.25 mg/kg/infusion) self-administration, the animals were divided into separate groups (*n* = 7–8) to undergo test procedures. Vehicle or OSU-6162 was administered during the 3 last cocaine self-administration session. Immediately after the last cocaine self-administration sessions, animals were either sacrificed (for biochemical analysis) or injected with pentobarbital and perfused intracardially (for IHC and in situ PLA analysis) (Fig. 1).

## Biochemical Binding Experiments

### Membrane Preparation

Frozen tissue was homogenized in ice-cold preparation buffer using a sonicator (Soniprep 150). The buffer contained 50 mM Tris-HCl, pH 7.4, 7 mM MgCl_2_, 1 mM EDTA, a cocktail of protease inhibitors (Roche Diagnostics, Mannheim, Germany), and 0.3 IU/ml adenosine deaminase (EC 3.5.4.4, Sigma-Aldrich). The membranes were precipitated by centrifugation at 4 °C for 40 min at 40,000 × *g* (Thermo scientific, Sorvall LYNX 6000, Stockholm, Sweden) and washed through re-homogenization in the same buffer once more. The protein concentration was determined for the membrane homogenates by means of BCA Protein Assay (Pierce, Sweden) using as a standard bovine serum albumin. Pelleted membranes were resuspended to a concentration of 0.15 mg/ml, immediately used or stored at− 80 °C until required.

### [3H]-raclopride Competition Binding Experiments

[3H]-raclopride binding was displaced by quinpirole to determine the proportion of receptors in the high-affinity state (RH), the high-affinity (Ki, High), and low-affinity (Ki, Low) values. Ventral striatum membrane preparations (60 μg protein/ml) were incubated with increasing concentrations of quinpirole (0.01 nM to 1 mM) and 2 nM [3H]-raclopride (75 Ci/mmol, Novandi Chemistry AB, Sweden) in 250 μl of incubation buffer (50 mM Tris-HCl, 100 mM NaCl, 7 mM MgCl2, 1 mM EDTA, 0.05% BSA, 1 mM DTT) and 0.3 IU/ml adenosine deaminase (EC 3.5.4.4, Sigma-Aldrich) for 90 min at 30 °C in the presence or absence of 100 nM of the A2AR agonist CGS-21680. Non-specific binding was defined by radioligand binding in the presence of 100 μM (+) – butaclamol (Sigma-Aldrich, Sweden). The incubation was terminated by rapid filtration using Whatman GF/B filters (Millipore Corp, Sweden) and a MultiScreenTM Vacuum Manifold 96-well followed by five washes (250 μl per wash) with ice-cold washing buffer (50 mM Tris-HCl pH 7.4). The filters were dried, 5 ml of scintillation cocktail was added, and the amount of bound ligand was determined after 12 h by liquid scintillation spectrometry.

### In Situ Proximity Ligation Assay (In Situ PLA)

To study the effects of OSU-6162, a Sigma1R ligand in low doses, on the A2AR-D2R heteroreceptor complexes densities changes after cocaine self-administration, the in situ PLA was performed as described previously (Borroto-Escuela et al. 2013; Borroto-Escuela et al. 2016; Borroto-Escuela et al. 2012). Free-floating formalin-fixed brain sections (30 μm-thick, cut using a cryostat) at Bregma level (1.0 0mm) from rats after cocaine self-administration were employed using the following primary antibodies: rabbit monoclonal anti-A2AR (AB1559F, 1:250; Millipore, Sweden), mouse monoclonal anti-D2R (MABN53, 1:600, Millipore, Sweden), and rabbit monoclonal anti-sigma1R (ab53852, 1:500, Abcam, Sweden). Primary antibodies were validated previously by means of immunohistochemistry in both rat brain tissue and HEK293 cell line ((Borroto-Escuela et al. 2017; Borroto-Escuela et al. 2018c; Feltmann et al. 2018)). Control experiments for in situ PLA procedures were performed in free-floating formalin-fixed rat brain sections employing only one primary antibody (mouse monoclonal anti-D2R (MABN53, 1:600, Millipore, Sweden). The in situ PLA analysis of this negative control showed 15.6% false-positive clusters compared to the positive control group value (100%). This false-positive signal was reduced even further (less than 4%) when the brain sections were kept in citric acid buffer for 45–60 min at 65 °C prior to the primary antibody incubation. Control experiments with similar results were also performed in cells transfected with cDNAs encoding only one type of receptor. The PLA signal was visualized and quantified by using a Leica TCS-SL SP5 confocal microscope (Leica, USA) and the Duolink Image Tool software. Briefly, fixed free-floating rat brain sections (storage at − 20 °C in Hoffman solution) were washed four times with PBS and quenched with 10 mM glycine buffer, for 20 min at room temperature. Then, after three PBS washes, incubation took place with a permeabilization buffer (10% fetal bovine serum (FBS) and 0.5% Triton X-100 or Tween 20 in Tris buffer saline (TBS), pH 7.4) for 30 min at room temperature. Again the sections were washed twice, 5 min each, with PBS at room temperature and incubated with the blocking buffer (0.2% BSA in PBS) for 30 min at room temperature. The brain sections were then incubated with the primary antibodies diluted in a suitable concentration in the blocking solution for 1–2 h at 37 °C or at 4 °C overnight. The day after, the sections were washed twice, and the proximity probe mixture (minus and plus probes, for details see Duolink instructions) was applied to the sample and incubated for 1 h at 37 °C in a humidity chamber. The unbound proximity probes were removed by washing the slides twice, 5 min each time, with blocking solution at room temperature under gentle agitation. The sections were incubated with the hybridization-ligation solution (BSA (250 g/ml), T4 DNA ligase (final concentration of 0.05 U/μl), Tween 20 (0.05%), NaCl 250 mM, ATP 1 mM, and the circularization or connector oligonucleotides (125–250 nM)) and incubated in a humidity chamber at 37 °C for 30 min. The excess of connector oligonucleotides was removed by washing twice, for 5 min each, with the washing buffer A (Sigma-Aldrich, Duolink Buffer A: 8.8 g NaCl, 1.2 g Tris Base, 0.5 ml Tween 20, dissolved in 800 ml high-purity water, pH to 7.4) at room temperature under gentle agitation and the rolling circle amplification buffer was added to the sections and incubated in a humidity chamber for 100 min at 37 °C. Then, the sections were incubated with the detection solution through hybridization (fluorescent oligonucleotide probes) in a humidity chamber at 37 °C for 30 min. In a last step, the sections were washed twice in the dark, for 10 min each, with the washing buffer B (Sigma-Aldrich, Duolink Buffer B: 5.84 g NaCl, 4.24 g Tris Base, 26.0 g Tris-HCl, dissolved in 500 ml high purity water, pH 7.5) at room temperature under gentle agitation. The free-floating sections were put on a microscope slide and a drop of appropriate mounting medium containing DAPI giving a blue staining of the nuclei (e.g., VectaShield or Dako) was applied. The cover slip was placed on the section and sealed with nail polish. The sections were protected against light and stored for several days at − 20 °C before confocal microscope analysis.

## Statistical Analysis

Data were analyzed using GraphPad Prism 5.0 (GraphPad Software Inc., San Diego, CA). All the data are shown as means ± SEM. In behavioral experiments, the number of responses on the active and inactive lever or the number of infusions was analyzed using a one-way analysis of variance (ANOVA) for repeated measurements, the latter analysis followed by post hoc Dunnett test. Data from competition experiments were analyzed by nonlinear regression analysis. The absolute values and the percent changes induced by A2A agonist CGS-21680 in the dopamine D2R high-affinity, low-affinity, and proportion of receptors in the high-affinity state were evaluated with paired Student’s *t*-test and nonparametric Mann-Whitney *U-*test, respectively. Data from in situ PLA experiments showing cluster density (clusters per nucleus per sampled field) were analyzed using a one-way ANOVA followed by post hoc Tukey’s test. The number of rats (*n*) in each experimental condition is indicated in figure legends. The *P* value 0.05 and lower was considered significant.

## Results

### Behavioral Analysis

Following 17 sessions, rats acquired cocaine self-administration (i.e., they received > 35 infusion/2 h under 0.25 mg/kg/infusion) and displayed < 10% variation in the number of infusions (Fig. 1A). The mean number of cocaine infusions per day during the last 6 self-administration days varied from 35 to 40. The total cocaine intake for vehicle group was 158 ± 28 mg/rat (means ± SEM of 7 rats) and for OSU-6162 group 156 ± 23 mg/rat (means ± SEM of 8 rats).

During the last 3 days of cocaine self-administration (0.25 mg/kg/infusion), the rats were pretreated with vehicle or OSU-6162 5 mg/kg.

OSU-6162 did not change the number of active and inactive lever presses [F(1, 26) = 1.094, p = 0.305] or the number of cocaine infusions after 1 h [F (1, 13) = 0.205, p = 0.657] and 2 h [F(1, 13) = 0.819, p = 0.382] (Fig. 1B).

No difference between the vehicle versus OSU-6162 groups for active and inactive lever presses or for infusions was observed on every treatment day (Fig. 1C).

## Studies with In Situ PLA on the Effects of OSU-6162 in Cocaine Self-Administration and Yoked-Saline Controls on D2R Heteroreceptor Complexes of Nucleus Accumbens with Focus on the D2R-Sigma1R Complexes

### D2R-Sigma1R Complexes

**Fig 2:**
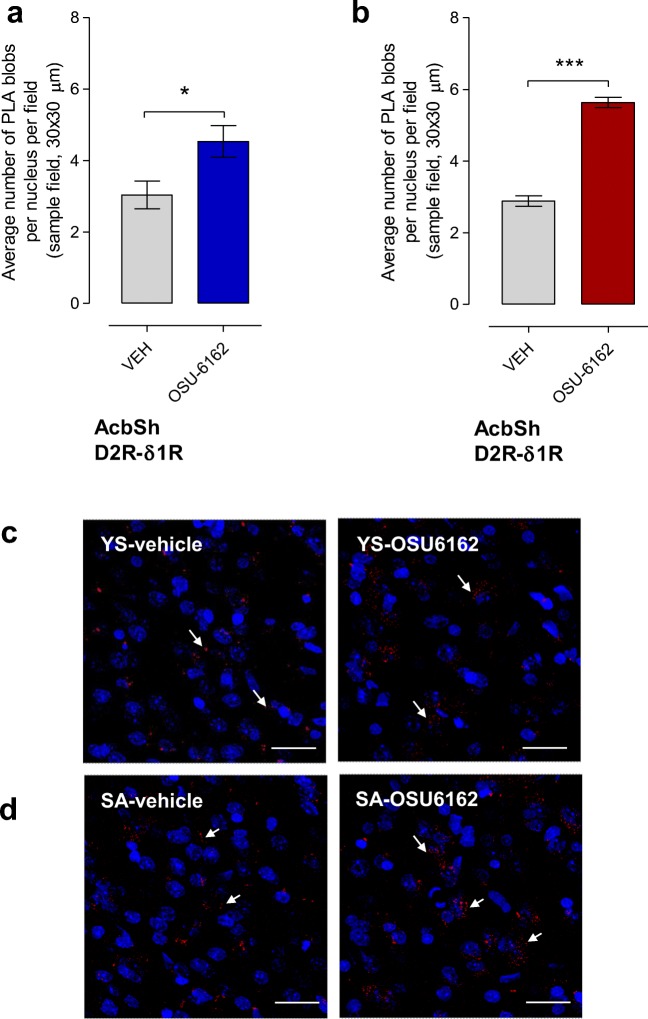
Effects of daily treatments for 3 days with OSU-6162 (OSU, 5 mg/kg, s.c.) compared with the corresponding daily vehicle (VEH) treatments on the density of PLA positive D2R-Sigma1R heteroreceptor complexes in the nucleus accumbens shell (AcbSh) in yoked-saline rats **(A)** and cocaine self-administration **(B)**. Means ± SEM(3–4 rats/group) are shown with OSU-6162 data in red. Student’s t-test (*p < 0.05), ns, not significant. Representative images of the red PLA positive complexes (white arrows) are presented in the nucleus accumbens shell (AcbSh) in yoked-saline rats **(C)** and cocaine self-administration **(D)**

In yoked-saline rats, the 3-day treatment with OSU-6162 (5 mg/kg) significantly increased (*p < 0.05) the density of the D2R-Sigma1R complexes in the nucleus accumbens shell versus the vehicle treated group (Figure 2A). A representative increase in the density of the red blobs in the shell of the nucleus accumbens is also presented comparing OSU-6162 and vehicle treated rats belonging to the yoked-saline group (Fig. 2C). In the cocaine self-administration rats, this OSU-6162 treatment caused a marked and highly significant increase in the density of D2R-Sigma1R heteroreceptor complexes in the nucleus accumbens shell (***P < 0.001) (Fig. 2B). The percent increase observed in the cocaine self-administration group after OSU-6162 treatment (5 mg/kg) was significantly different versus the percent increase observed in the yoked-saline group after the OSU-6162 treatment. Thus, the statistical analysis demonstrates that the percent values are significantly different from each other. Mann-Whitney U test, Mean ± SEM, 4 rats/group, *p < 0.05. Cocaine self-administration (216.0 ± 18.7%) vs yoked-saline (153.5 ± 24.2%). A representative increase in the density of PLA blobs in the shell of the nucleus accumbens is also presented in the cocaine self-administration group (Fig. 2D).

### A2AR-D2R Heteroreceptor Complexes

**Fig. 3:**
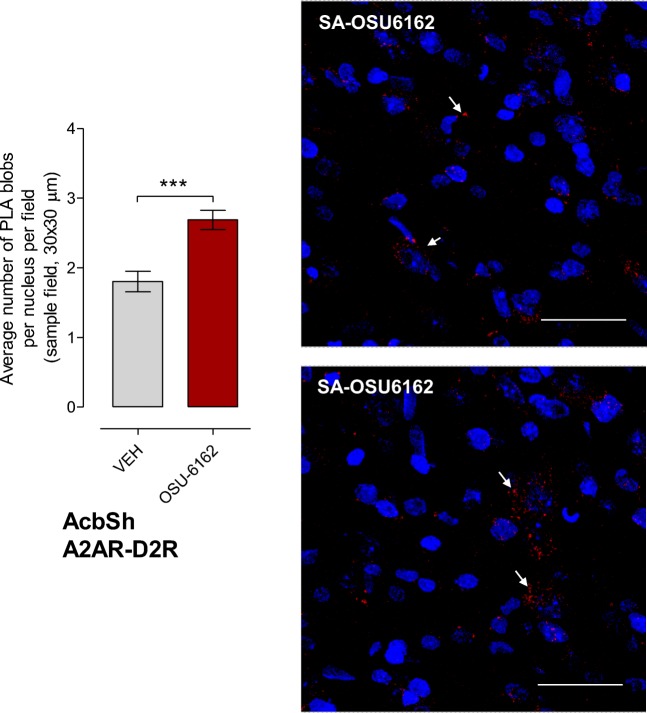
Effects of daily treatments for 3 days with OSU-6162 (OSU, 5 mg/kg, s.c.) compared with the corresponding daily vehicle (VEH) treatments on the density of PLA positive A2AR-D2R heteroreceptor complexes in the nucleus accumbens shell (AcbSh) in cocaine self-administration. Means ± SEM (4 rats) are shown with OSU-6162 data in red. Student’s t-test (***p < 0.001). Representative images of the red PLA positive A2AR-D2R heteroreceptor complexes ( white arrows) are presented in AcbSh.

For comparison, also the A2AR-D2R complexes were studied in cocaine self-administration. Daily treatment for 3 days with OSU-6162 (5 mg/kg, s.c.) produced a highly significant increase also in the density of these heteroreceptor complexes in the nucleus accumbens shell versus the vehicle-treated group in cocaine self-administration. (Fig. 3).

## Effects of OSU-6162 Treatment on the Allosteric A2AR-D2R Receptor-Receptor Interactions in the Ventral Striatum in Cocaine Self-Administration

**Fig. 4:**
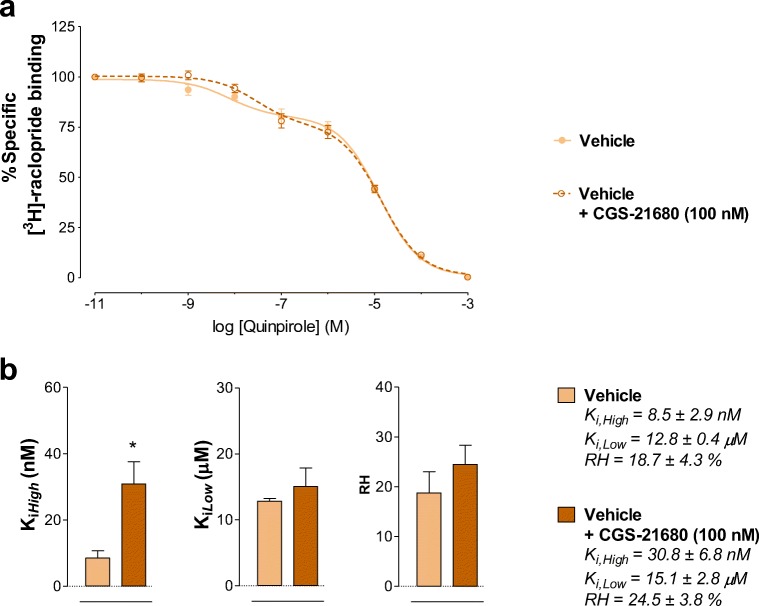
[^3^H]-raclopride/quinpirole competition experiments to determine changes in D2R affinities induced by adenosine A2AR agonist CGS-21680 in the yoked-saline (vehicle) rat group (control group). (**A**) Competition experiments involving dopamine D2-likeR antagonist [^3^H]-raclopride binding versus increasing concentrations of quinpirole were performed in ventral striatal membrane preparations from control group (60 μg/ml) in the presence or absence of the adenosine A2A agonist CGS-21680 (100 nM) as indicated. Nonspecific binding was defined as the binding in the presence of 100 μM (+) – butaclamol. (**A**) [^3^H]-raclopride/quinpirole displacement curve based on the values of four rats with each experiment performed in duplicate. The binding values are given in percent of specific binding at the lowest concentration of quinpirole employed. (**B**) Analysis and presentation are given of the A2AR agonist CGS-21680 (100 nM) induced changes in the high-affinity value (K_*i, High*_), low-affinity value (K_*i, Low*_), and the value on the proportion of receptors in the high-affinity state (RH). Means ± SEM. are given from four independent experiments performed in duplicate. Statistical analysis was performed by paired Student’s t-test, *(*p* < 0.05): the group of rats treated with CGS-21680 is significantly different compared to the group receiving only the saline solution

In maintenance of cocaine self-administration, the A2AR agonist CGS-21680 (100 nM) ex vivo produced a significant reduction of the affinity of the D2R high-affinity agonist binding sites compared to vehicle ex vivo (*p = 0.0424, paired Student’s *t*-test) (Figure 4 A, B). No significant effects were induced by CGS-21680 ex vivo on the K_*i, Low*_ and RH values (Figure 4B).

**Fig. 5:**
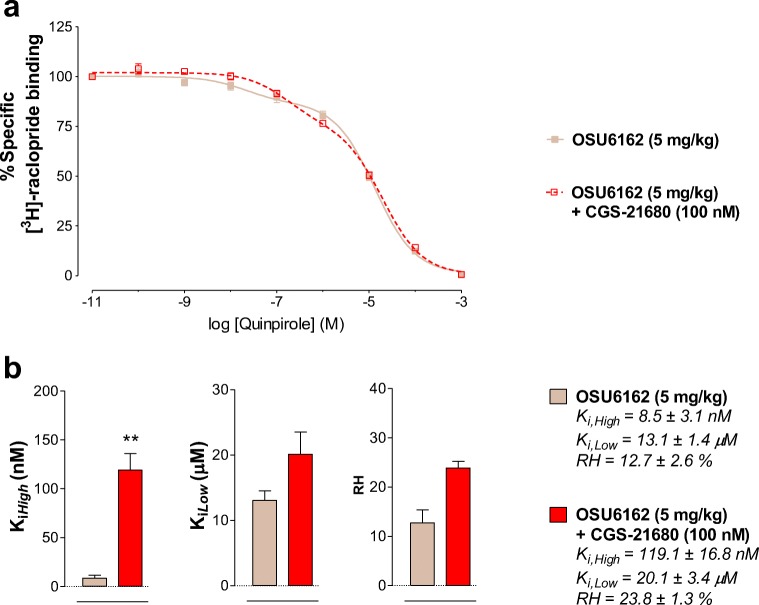
[^3^H]-raclopride/quinpirole competition experiments to determine changes in D2R affinities induced by adenosine A2AR agonist CGS-21680 in the cocaine self-administration rat group. (**A**) Competition experiments involving dopamine D2-likeR antagonist [^3^H]-raclopride binding versus increasing concentrations of quinpirole were performed in ventral striatal membrane preparations from cocaine self-administration rat group (60 μg/ml) in the presence or absence of the adenosine A2A agonist CGS-21680 (100 nM) as indicated. Nonspecific binding was defined as the binding in the presence of 100 μM (+) – butaclamol. (**A**) [^3^H]-raclopride/quinpirole displacement curve based on the values of four rats with each experiment performed in duplicate. The binding values are given in percent of specific binding at the lowest concentration of quinpirole employed. (**B**) Analysis and presentation are given of the A2AR agonist CGS-21680 (100 nM) induced changes in the high-affinity value (K_*i, High*_), low-affinity value (K_*i, Low*_), and the value on the proportion of receptors in the high-affinity state (RH). Means ± SEM. are given from four independent experiments performed in duplicate. Statistical analysis was performed by paired Student’s t-test. **(*p* < 0.01): the group of rats treated with CGS-21680 is significantly different compared to the group receiving saline-solution

**Fig. 6:**
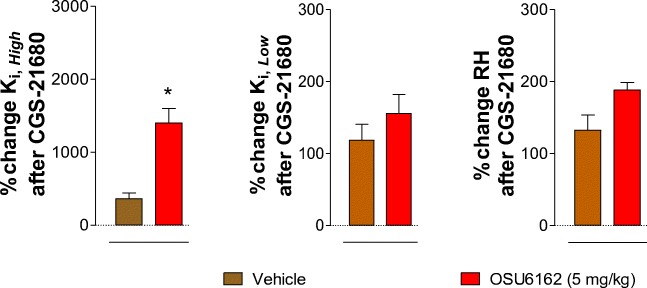
Analysis and presentation of the A2A agonist CGS-21680 (100 nM) induced percent changes in D2R binding comparing the yoked-saline group and cocaine self-administration group (given in % of values in the absence of CGS-21680) with regard to the dopamine D2R high-affinity value (K_*i, High*_), the low-affinity value (K_*i, Low*_), and the value on the proportion of receptors in the high-affinity state (RH). Means ± SEM. are given for four independent experiments performed in duplicate. Statistical analysis was performed by nonparametric Mann-Whitney *U-*test. *(*p* < 0.05): cocaine self-administration group is significantly different compared to the yoked-saline group

In the absence of CGS-21680 ex vivo, the 3-day OSU-6162 treatment at 5 mg/kg in maintenance of cocaine self-administration failed to significantly alter the D2R K_*i, High*_ values versus the vehicle-treated cocaine self-administration group (Fig. 4B, 5B). However, in presence of CGS-21680 (100 nM) ex vivo, the OSU-6162 treatment markedly enhanced the ability of the A2AR agonist to increase the D2R K_*i, High*_ values (**p = 0.0082, paired Student’s *t*-test) (Fig. 5B). The percent change obtained in the D2R K_*i, High*_ values in the OSU-6162 treated group versus the vehicle-treated cocaine self-administration group was significant (*p < 0.0286, nonparametric Mann Whitney *U*-test) (Fig. 6B). However, no significant effects were induced by combined treatment with OSU-6162 (in vivo) and CGS-21680 (ex vivo) in the K_*i, Low*_ and RH values versus treatment with OSU-6162 alone (Fig. 5 B).

## Discussion

It was previously demonstrated that cocaine self-administration in the rat causes a selective increase in the antagonistic allosteric A2A-D2R interactions in ventral striatum (Pintsuk et al. 2016) versus the yoked-saline group. The above increase in the A2AR-D2R receptor interactions was associated with an increase in the A2AR-D2R and the D2R-Sigma1R heteroreceptor complexes in the nucleus accumbens shell (Borroto-Escuela et al. 2019; Borroto-Escuela et al. 2018b). Thus, maintenance of cocaine self-administration appears capable of favoring the formation of A2AR-D2R and D2R-Sigma1R heteroreceptor complexes from A2AR and D2R and D2R and Sigma1R homomers/monomers, respectively, in this region. These results are also compatible with an increased formation of trimeric A2AR-D2R-Sigma1R heteroreceptor complexes in this area. It was proposed that long term cocaine use can produce pathological A2AR-D2R-Sigma1R complexes which can lead to a permanent and marked brake on the D2R protomer function resulting in development of cocaine addiction (Borroto-Escuela et al. 2018b). Treatment should involve removal of this pathological and permanent heteroreceptor complexes with suppressed Gi/o-mediated D2R signaling. Options to reduce cocaine addiction could be the use of brain-penetrant-interfering peptides that disrupt the A2AR-D2R-Sigma1R complex or brain-penetrant heterobivalent drugs with D2R agonist and A2AR antagonist pharmacophores to reduce the D2R brake in the A2AR-D2R-Sigma1R complexes (Borroto-Escuela et al. 2018b). It is certainly possible to also use to treat cocaine addiction the A2AR antagonists used in treatment of Parkinson’s disease like istradefylline (Nourianz) (Borroto-Escuela and Fuxe 2019; Borroto-Escuela et al. 2018a; Fuxe and Borroto-Escuela 2019).

It is a highly interesting that A2AR activation primarily allosterically modulates the high-affinity state of the D2R protomer in the A2AR-D2R heteromer which is the most relevant functional state (Briand et al. 2008; Pintsuk et al. 2016; Seeman 2011). Thus, the antagonistic A2AR-D2R interaction may not involve antagonism of D2R recognition in the low-affinity state. As a consequence, it may be more difficult for A2A receptor agonists to antagonize the effects of high concentrations of DA in the brain, which activates also the low-affinity state of the D2R protomer, at least with regard to D2R protomer recognition.

The major neurochemical result obtained in the current paper would agree with this proposal. Thus, in cocaine self-administration, the monoamine stabilizer OSU-6162 (Carlsson and Carlsson, 2004; Steensland et al. 2012) in a low dose of 5 mg/kg mainly affects the Sigma1R for which it has nanomolar affinity (Sahlholm et al. 2013). Following a 3-day treatment with this dose, OSU-6162 significantly enhanced the ability of cocaine self-administration to increase the formation of D2R-Sigma1R heteroreceptor complexes in the nucleus accumbens shell. In line with previous work, OSU-6162 versus vehicle treatment also enhanced the formation of A2AR-D2R complexes in cocaine self-administration in nucleus accumbens shell.

Of high interest is also that the A2AR agonist CGS-21680 (100 nM) when added ex vivo to membrane preparations of the ventral striatum after in vivo treatment with OSU-6162, as above, significantly and markedly reduced the affinity of the high-affinity D2R agonist-binding sites. This action of CGS 21680 ex vivo was reduced in the vehicle treated rats in which a diminished reduction of the affinity of the D2R high-affinity agonist-binding sites was observed. Thus, the inhibitory A2AR-D2R interactions can be strengthened by the A2AR agonist activation after pretreatment with monoamine stabilizer in a dose mainly targeting the Sigma1R.

It appears as if the major action of the A2AR agonist, unlike the action of the low dose of the monoamine stabilizer, is to increase the antagonistic allosteric A2AR-D2R interactions through activation of the A2AR protomer. Instead actions at the Sigma1R by OSU-6162 can favor the formation of the receptor complexes studied due to enhancement of cocaine-induced expression of the Sigma1R and Sigma1R-induced increases in the affinity of the A2AR and D2R receptors for each other. Taken together, the neurochemical analysis performed would also be compatible with an increased formation of trimeric A2AR-D2R-Sigma1R complexes with an enhanced allosteric brake on Gi/o-mediated function of the D2R protomer (Borroto-Escuela et al. 2018b).

It is difficult to know the behavioral relevance of the neurochemical changes observed after a low-dose OSU-6162 treatment. No significant effects were observed with the 3-day treatment with OSU-6162 alone on the active lever presses for cocaine and on number of cocaine infusions over 2 h per day. However, a modest non-significant reduction was observed of cocaine self-administration on day 3 of OSU-6162 treatment. It opens up the possibility of the existence of A2AR-D2R-Sigma1R complexes in the ventral striatum (Borroto-Escuela et al. 2018b) in which the Sigma1R upon activation by cocaine and OSU-6162 may enhance the antagonistic allosteric A2AR-D2R interactions in this trimeric complex.

The non-significant reduction observed with OSU-6162 of cocaine self-administration may be related to the existence of a significant number of D2R-Sigma1R complexes in nucleus accumbens in which cocaine and OSU-6162 enhance D2R recognition and signaling (Borroto-Escuela, 2019). It should also be considered that in spite of the low dose used with limited binding to the D2R, OSU-6162 may exert minor effects on the D2R through partial D2R agonist actions (Fredriksson et al. 2018). In addition, cocaine and OSU-6162 in vivo may also enhance D1R signaling in Sigma1R-D1R complexes (Navarro et al. 2010) of the accumbal reward GABA pathway. Such actions will also favor cocaine self-administration. It becomes clear that more work is necessary to obtain evidence for the hypothesis that enhanced antagonistic A2AR-D2R receptor-receptor interactions in A2AR-D2R-Sigma1R complexes and can be a major mechanism for development of cocaine addiction due to a permanent brake on D2R protomer signaling over Gi/o.

In conclusion, the neurochemical results suggest that a low dose of the monoamine stabilizer OSU-6162 targeting the Sigma1R (Sahlholm et al. 2013) may significantly enhance the increase in A2AR-D2R and Sigma1R-D2R complexes in the nucleus accumbens shell observed in cocaine self-administration. The increase in the A2A-D2R heteroreceptor complexes induced by cocaine and OSU-6162 can be associated with a clear-cut enhancement of the antagonistic A2AR-D2R interactions in the ventral striatum. Their behavioral relevance remains to be established but the results are compatible with a role of A2AR-D2R-Sigma1R in cocaine addiction through allosteric inhibition of the D2R protomer recognition and Gi/o-mediated signaling.
